# Cytokine profiling in healthy children shows association of age with cytokine concentrations

**DOI:** 10.1038/s41598-017-17865-2

**Published:** 2017-12-19

**Authors:** Marie-Luise Decker, Verena Gotta, Sven Wellmann, Nicole Ritz

**Affiliations:** 1 Infectious Diseases and Vaccinology Unit, University of Basel Children’s Hospital, 4056 Basel, Switzerland; 2 Paediatric Pharmacology and Pharmacometrics, University of Basel Children’s Hospital, 4056 Basel, Switzerland; 3 Neonatology Unit, University of Basel Children’s Hospital (UKBB), 4056 Basel, Switzerland

## Abstract

Cytokine-based diagnostic assays are increasingly used in research and clinical practice. Assays developed for adults such as the interferon-gamma release assay for tuberculosis show inferior performance in children. Limited evidence suggests that release of cytokines is influenced by age but normal ranges of cytokines in children are lacking. Whole blood of healthy children (0–12 years) undergoing elective/diagnostic procedures was stimulated with SEB, PHA, *Candida albicans* for 24 hours or left unstimulated. Concentrations of eight cytokines were measured by multiplex bead-based immunoassays and associations with age and other factors quantified by regression analysis. 271 children (median age 5.2 years) were included. In unstimulated samples IL-1ra, IP-10 and TNF-α concentrations decreased by up to −60% with age. Following antigen stimulation, an age-associated increase (ranging from +90% to +500%) was observed for all cytokines except IL-1ra (significant for IL-4, IFN-γ and TNF-α). Inter-individual variability in cytokine concentrations was large with a coefficient of variation ranging from 42% to 1412%. Despite inter-individual variation age was identified as a strong influencing factor of cytokine concentrations. Age-specific normal values need to be considered for cytokine-based diagnostic purposes. These results are relevant for development of novel cytokine-based diagnostic assays and for optimal dosing of therapeutic agents targeting cytokines.

## Introduction

Cytokines are soluble proteins released by a broad range of cell types particularly including the innate and adaptive immune system. They are involved in the regulation of complex processes such as proliferation, differentiation and inflammation. Their mode of action is only partially understood since they frequently act in highly complex networks referred to as the “cytokine milieu” and their function may be dependent on target cells and location^1^.

The detection of cytokines continues to attract considerable attention as a result of their importance in key signalling processes. Cytokine concentrations are therefore measured in many body fluids, tissues and cells for diagnostic or prognostic purposes. The number of diseases and conditions in which cytokine signatures are measured is increasing and cytokine-based disease taxonomy is starting to be proposed for a number of chronic inflammatory conditions including rheumatoid arthritis, juvenile idiopathic arthritis, inflammatory bowel disease, psoriasis and multiple sclerosis^2^. In addition, for acute infections the measurement of cytokines may help to confirm or rule out severe bacterial infections such as sepsis and community acquired pneumonia in the paediatric and adult setting^3,4^ In paediatrics the use of cytokines for diagnostic purposes has most commonly been studied in neonatal sepsis, tuberculosis, pneumonia, neuroinflammatory diseases and hemophagocytic lymphohistiocytosis^5,6^.

Despite accumulating evidence of the importance of cytokines in infectious, chronic inflammatory and neoplastic diseases very few cytokine-based tests are currently used in clinical routine. One test which has been used for more than a decade is the interferon (IFN)-γ release assay (IGRAs) for the diagnosis of tuberculosis. This test detects - in contrast to the non-specific measurement of cytokines – *M. tuberculosis*-specific IFN-γ^7,8^. IGRAs have been developed for adults and use in children shows consistently poorer performance^9,10^. One explanation is that age influences the capacity to produce IFN-γ, which has been shown in studies investigating age-associations of results of IGRAs in children and adults^9,11^. Further evidence on the influence of age on cytokine release is provided by studies investigating IFN-γ-producing T cells in healthy children, adolescents and adults, showing a progressive increase until early teenage years^12,13^. Information on development of cytokine release is therefore critical for the interpretation and understanding of any cytokine-associated assay currently used in the paediatric population. In addition, research and development of diagnostic tests in children using cytokines including viral respiratory infections, asthma, autoinflammatory diseases and autism spectrum disorders is advancing, however, knowledge on normal development of cytokine expression and normal values of cytokine concentrations in healthy individuals is very limited^14–17^. The aim of this study was to assess cytokine concentrations in healthy children, to provide age-dependent normal ranges in children and to determine other potential factors influencing cytokine concentrations.

## Material and Methods

### Study population

Healthy children undergoing elective surgery or interventions at the University of Basel Children’s Hospital between December 2013 and October 2015 were eligible for inclusion. Children up to 12 years of age were approached and informed consent was obtained from parents at least one day prior to the intervention. The following exclusion criteria were used: history of acute disease and/or fever in the prior four weeks; immunisation administered in the prior two months; known immunodeficiency, haemato-oncological or autoimmune disease; current or previous immunomodulatory medication (including oral corticosteroids but excluding inhaled corticosteroids). The study was approved by the ethics committee of North-West Switzerland (Number: 162/13) and all methods were performed in accordance with the relevant guidelines and regulations.

After informed consent was obtained information for each child was collected in a standardised case report form and de-identified data was added to a password secured database (Epidata manager, v.1.4.4.4 and Epidata entry client, v.1.4.4.2 by EpiData Association, Denmark). The following patient details were recorded: age, sex, weight and height, previous medical history, current and previous medication (including vitamins or dietary supplements), dietary habits (including fruit vegetable and fish intake) second-hand smoke exposure and exposure and presence of pets. Time and day of the blood collection, type of elective intervention and anaesthetic drug used were also recorded.

### Sample preparation and stimulation

A heparinised venous blood sample was collected at the beginning of anaesthesia. All samples were processed within two hours of blood collection. Whole blood was stimulated at 37 °C for 24 hours with phytohaemagglutinin (PHA, Merck chemicals LTD., Beeston, Nottingham, UK) at a concentration of 5 µg/ml, staphylococcus enterotoxin B (SEB, Sigma Aldrich GmbH, Schnelldorf, Germany) at a concentration of 10 µg/ml, *Candida albicans* (Greer Laboratories, Lenoir, USA) at a concentration of 4 µl/ml or left unstimulated (nil control) in the presence of CD 28 and CD 49d antibodies (Biolegend Inc., San Diego, CA 92121, USA). After stimulation supernatants were stored at −80 °C until further analysis. Short-term whole blood stimulation assays of fresh blood were used as these require small blood volumes, and show increased sensitivity and lower background as opposed to assays using peripheral blood mononuclear cells^18,19^. For Vitamin D measurement, whole blood was centrifuged at 1000 G for 5 minutes and plasma stored at −80 °C until later batched analysis.

### Cytokine measurement

Interleukin (IL)-1ra, IL-2, IL-4, IL-6, IL-10, IFN-γ, tumor necrosis factor (TNF)-α and IFN-γ-inducible protein (IP)-10 were determined using a human cytokine/chemokine magnetic bead panel (Milliplex MAP kit, Millipore Corp., Billerica, MA, USA) and measured by a Magpix Luminex instrument and xponent software (version 4.2, Luminex Corp, Austin, Texas, US) according to manufacturer’s instruction. Based on previous optimisation experiments SEB- and PHA-stimulated samples were analysed in a 1:10 dilution, *C. albicans-*stimulated samples and nil control samples were analysed undiluted. The overall intra- and inter- assay precision is reported by the manufacturer as 2–19%, and accuracy as 87–107% over the calibration range of 3.2–10’000 pg/mL cytokine concentration^20^.

### Vitamin D analysis

25-hydroxy-vitamin D3 concentrations were analysed in the chemistry core laboratory at the University of Basel Hospital with a Chemiluminescence Immunoassay (CLIA) method using a Liaison instrument by DiaSorin.

### Statistical analyses

The associations of cytokine concentrations with age, body weight for age (z-score)^21^ sex and vitamin D were assessed using univariate censored linear regression analysis on log-transformed concentrations (details on the linear regression model in supplementary material box 1): cytokine concentrations below the lower limit of quantification (LLOQ) of 3.2 pg/mL were left-censored, while cytokine concentrations above the upper limit of quantification (ULOQ) of 10’000 pg/mL were extrapolated from the analytical calibration curve. All significant associations defined as parameter estimates with a p-value < 0.05 were reported; no p-value correction was performed for multiple testing. Kendall rank correlation coefficient was used to estimate associations of age with body weight for age (z-score) sex and vitamin D. The associations of log-transformed cytokine concentrations with other confounding factors (including fruit, vegetable, fish intake and secondhand smoke exposure) were screened using scatter- and boxplots. Statistical analysis was performed were visual screening indicated differences. All statistical analyses and figures were done using R (version 3.2.1; R Development Core Team, Vienna, Austria, http://www.r-project.org).

As age-association was the main variable of interest between-subject variability and alternative relationships (including linear, exponential and logistic functions) of cytokine concentrations with age were further assessed using four models (the structural models and selection criteria are detailed in supplementary material box 2). For this purpose a mixed-effect censored regression analysis was performed including stimulated and unstimulated pooled data of each cytokine in a multivariate outcome (NONMEM software, version 7.3.0; Icon Development Solutions, Ellicott City, MD, USA, executed using Pearl-speaks-NONMEM scripts PsN version 3.7.6, http://psn.sourceforge.net). Individual random effect models and residual variability models are described in supplementary material box 2 in detail. The model fit of competing models was compared in simulation based diagnostics (visual predictive check). The final models were used to simulate the predicted age-dependent median with the 5^th^ and 95^th^ percentiles (details on calculations of the percentiles in supplementary material box 3).

### Data availability statement

The datasets generated and analysed during the current study are available from the corresponding author on reasonable request.

## Results

A total of 392 parents agreed for their children to take part in the study, out of which 271 met the final inclusion criteria. Reasons for exclusion after recruitment included: a history of acute disease or fever (in past month) or vaccination (in the past two months) (n = 86), failed blood collection (n = 35). Demographic details and other characteristics of the study population are summarised in Table 1.

**Table 1:** Demographic and other characteristics of the 271 children included in the study.

Characteristic	Result
**Age**	
range (years)	0.1–12.8
median (IQR^a^) (years)	5.3 (3.5–7.9)
**Body weight**	
range (kg)	4.1–74.0
median (IQR) (kg)	19.6 (15.0–26.8)
z-score^b^ (SD^c^) (kg)	0.3 (−0.5–1.0)
**Sex**	
boys (%)	74
**Vitamin D**	
range (nmol/L)	11–142
median (IQR) concentration (nmol/L)	54 (40–69)
**Type of elective intervention**	
circumcision (%)	32
adenoidectomy/paracentesis (%)	21
orchidopexy/hernia repair (%)	17
other* (%)	30
**Anaesthetic drug used**	
sevoflurane and nitrous oxide (%)	89
sevoflurane (%)	9
intravenous thiopental/propofol (%)	2
**Second-hand smoke exposure**	
none (%)	64
one family member (%)	25
both parents (%)	11
**Daily fruit and vegetable intake**	
0–1 portion (%)	24
2–4 portions (%)	69
>5 portions (%)	7
**Weekly fish intake**	
None (%)	37
<1 portion (%)	24
≥ 1 portion (%)	39

^a^IQR = inter-quartile range, ^b^z-score = body weight for age, ^c^SD = standard deviation.

*Including: removal of osteosynthetic material, gastroscopy, colonoscopy, deflux injection.

### Association of cytokines with anthropometric and other variables

For unstimulated samples ≥69% of results were below the LLOQ for the following cytokines: IL-2, IL-4, IL-10 and IL-6 (Table 2). Only IP-10 concentrations were above the ULOQ in 3% of the results (Table 2). For stimulated samples, up to 22% of results were below the LLOQ depending on the stimulatory antigen used (Table 2). Almost all results were below the ULOQ except for IP-10 which was above ULOQ in 46–88% depending of the stimulatory antigen used. A numerical summary of measured cytokine concentrations is given in Table 2.

**Table 2:** Summary of cytokine concentrations measured in 271 healthy children. Calibration range was 3.2–10’000 pg/ml for unstimulated and *C. albicans*-stimulated samples, and 32–100’000 pg/mL for SEB- and PHA-stimulated samples. *Concentrations above the limit of quantification were extrapolated from the calibration curve.

Cytokine	Number (%) <LLOQ^a^	Number (%) >ULOQ^b^	Median (pg/mL)	Min (pg/mL)	Max (pg/mL)	P25 (pg/mL)	P75 (pg/mL)
**Unstimulated**							
IL-1ra	18 (7)	0	76	<3.2	1524	34	162
IL-2	246 (91)	0	2	<3.2	1047	<3.2	<3.2
IL-4	224 (83)	0	2	<3.2	559	<3.2	<3.2
IL-6	188 (69)	0	2	<3.2	768	<3.2	<3.2
IL-10	223 (82)	0	2	<3.2	247	<3.2	<3.2
IFN-γ	106 (39)	0	5	<3.2	535	<3.2	14
IP-10^*^	1 (0)	8 (3)	905	<3.2	61760	645	1528
TNF-α	17 (6)	0	10	<3.2	485	7	14
**SEB** ^**c**^							
IL-1ra	5 (2)	0	1312	<32	10742	864	2063
IL-2*	1 (0)	1 (0)	20173	<32	1101156	14195	27361
IL-4	2 (1)	0	880	<32	24641	447	1576
IL-6	1 (0)	0	3368	<32	13046	1762	5237
IL-10	1 (0)	0	3058	<32	18098	1899	4957
IFN-γ*	1 (0)	17 (6)	28963	<32	348112	13484	49364
IP-10*	0	238 (88)	191911	11098	1717241	137147	255704
TNF-α	0	0	7903	33	30503	4995	10826
**PHA** ^**d**^							
IL-1ra	20 (7)	0	643	<32	9444	351	1059
IL-2	26 (10)	0	226	<32	9472	85	570
IL-4	14 (5)	0	960	<32	29274	389	2031
IL-6	16 (6)	0	1776	<32	19944	844	3198
IL-10	10 (4)	0	374	<32	3470	173	725
IFN-γ	4 (1)	0	771	<32	9507	362	1401
IP-10*	0	140 (52)	102853	894	9613370	59012	162812
TNF-α	5 (2)	0	553	<32	7696	295	1278
***C. albicans***							
IL-1ra	5 (2)	0	547	<3.2	4852	147	1234
IL-2*	18 (7)	1 (0)	255	<3.2	14294	47	647
IL-4	59 (22)	0	39	<3.2	1931	7	94
IL-6*	42 (15)	1 (0)	64	<3.2	15306	8	342
IL-10	47 (17)	0	17	<3.2	3520	6	35
IFN-γ	15 (6)	0	40	<3.2	4057	15	97
IP-10*	0	125 (46)	8764	192	127699	3690	18571
TNF-α	4 (1)	0	36	<3.2	1468	16	81

^a^LLOQ = lower limit of quantification, ^b^ULOQ = upper limit of quantification, ^c^SEB = Staphylococcal enterotoxin B, ^d^PHA = Phytohaemagglutinin.

**Figure 1:**
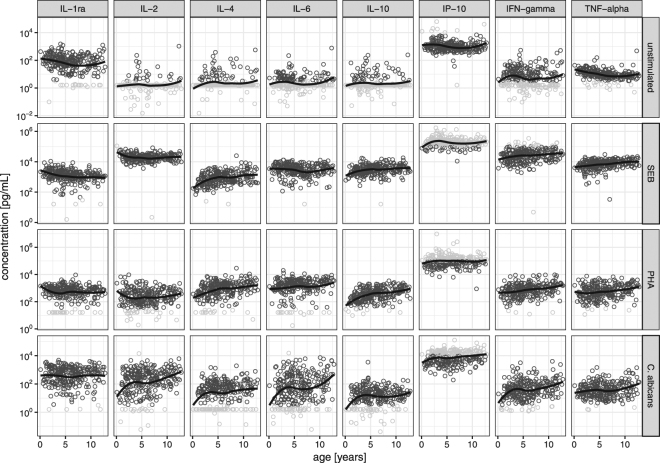
Observed cytokine concentrations over age on a semi-logarithmic scale (normal-scale for age and a log10-scale for cytokines). Eg concentration of 10^−2^ pg/mL equals a concentration of 0.01 pg/mL, 10^0^ equals 1 pg/mL, 10^2^ equals 100 pg/mL (note that the depicted range of concentration differs between stimulatory antigens used for better readability). *Dark grey circles*: concentrations measured within the range calibration curve of the analytical method. *Light grey circles:* concentrations beyond the calibration curve limits, i.e. below or above the limit of quantification. *Black line*: non-parametric regression line (locally weighted smoothing).

**Figure 2:**
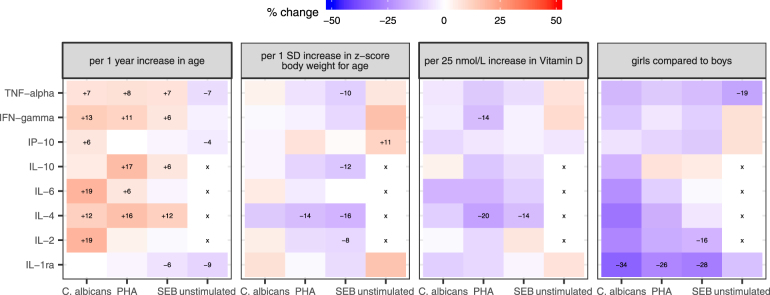
Association of cytokine levels with age, body weight (z-score body weight for age), vitamin D concentrations and sex. The color indicates the general direction of association (red = increase, blue = decrease). The depicted numerical percentage (%) change per indicated unit was estimated from censored linear regression on log-transformed cytokine concentrations and is depicted for significant associations only (regression coefficient with a p-value < 0.05). X: no regression analysis performed since >50% of data below limit of quantification.

Age-associated changes in cytokine concentrations were found for several cytokines and stimulatory antigens used. In unstimulated samples a decrease of cytokine concentration with age was seen for IL-1ra, IP-10 and TNF-α (Fig. 1) and calculated to be between −9% to −4% per year of age (Fig. 2). Contrary to this, in stimulated samples IL-4, IFN-γ and TNF-α showed an increase with age for all stimulatory conditions (Figs 1 and 2). In addition, in *C. albicans*-stimulated samples an increase of cytokine concentration was seen for further cytokines measured including IL-2, IL-6 and IP-10, with a calculated increase of +7 to +19% per year of age (Figs 1 and 2).

Body weight for age (z-score) was associated with changes of cytokine concentrations in few instances only. An increase in cytokine concentration was seen with a higher z-score for IFN-γ, IL-1ra, IP-10 and TNF-α, which reached statistical significance only for IP-10 in unstimulated samples (Fig. 2). Conversely, in stimulated samples an increase of the z-score was associated with a decrease of IL-4 concentrations independent of the stimulatory antigen used and a decrease of IL-2, IL-10 and TNF-α concentrations in SEB-stimulated samples (Fig. 2).

Analysis of sex showed that cytokine concentrations were generally lower in girls compared to boys. This difference reached statistical significance for TNF-α in unstimulated samples, for IL-1ra independent of the stimulatory antigen used and for IL-2 in SEB stimulated samples (Fig. 2).

Concentrations of 25-hyroxy-vitamin D3 had less influence on cytokine concentrations compared to age, z-score and sex. An increase of 25-hyroxy-vitamin D3 concentration measured in serum was significantly associated with a decrease of IL-4 in SEB- and PHA-stimulated samples and of IFN-γ in PHA-stimulated stimulated samples only (Fig. 2).

Correlations of age with weight for age (z-score), vitamin D3 and sex showed weak negative correlations for children <5 years for weight for age (z-score) (Kendall rank correlation coefficient: −0.08) and for vitamin D (Kendall rank correlation coefficient: −0.13) and a weak positive correlation for age with girls median age being 6.5 years versus 4.8 years in boys (Kendall rank correlation coefficient: +0.16).

For the remaining parameters including time of the day of blood collection, type of elective intervention, anesthetic drug used, dietary habits, supplement intake, second hand smoke exposure and presence of pets, no associations or trends were seen (data not shown).

### Further characterization of association of age with cytokine concentrations

An exponential function (model 2, see supplementary material Box 2) best described the relationship of age with cytokine concentrations in most instances. For two conditions (unstimulated TNF-α and SEB-stimulated IL-2 concentrations) a physiologic asymptote with increasing age (model 3, see supplementary material Box 2) was additionally quantifiable and improved the data description. The expected cytokine concentration in infancy (β_0_) and at 12 years of age (calculated from the best model, e.g. for model 2 by β_0_∙exp(β_1_∙12 years)) are shown in Table 3 (further details on all model parameter estimates are shown in supplementary material Table 1).

**Table 3:** Summary of expected cytokine concentrations in infancy and at 12 years of age and changes and coefficient of variation of estimated inter-individual variability.

Cytokine	Expected value in infancy (ng/ml)	Expected value for 12 years of age (ng/ml)	Change over 12 years (%)	Inter-individual variability (%)
**unstimulated**				
IL-1ra	97	40	−58	244
IL-2	<3.2	<3.2	NA^a^	NA
IL-4	<3.2	<3.2	NA	NA
IL-6	<3.2	<3.2	NA	NA
IL-10	<3.2	<3.2	NA	NA
IFN-γ	5.4	5.4	—	248
IP-10*	1071	1071	—	101
TNF-α	19.5	7.5	−62	73
**SEB** ^**b**^				
IL-1ra	1666	801	−52	123
IL-2*	46834	19301	−59	42
IL-4	458	1614	+253	111
IL-6	2847	2847	—	114
IL-10	2190	4243	+93	76
IFN-γ*	19200	38503	+101	104
IP-10*	190133	190133	—	62
TNF-α	4740	10859	+129	66
**PHA** ^**c**^				
IL-1ra	505	505	—	175
IL-2	225	225	—	217
IL-4	358	1829	+411	201
IL-6	1263	1263	—	260
IL-10	146	844	+477	130
IFN-γ	413	1340	+224	135
IP-10*	97973	97973	—	111
TNF-α	352	930	+164	157
**C. albicans**				
IL-1ra	372	372	—	327
IL-2*	65	392	+505	641
IL-4	15	49	+220	437
IL-6*	22	130	+491	1412
IL-10	15	15	—	206
IFN-γ	22	83	+274	132
IP-10*	5601	11235	+101	266
TNF-α	26	57	+116	169

^a^NA = not applicable (Data not analysed since over 69% of samples below the lower limit of quantification), ^b^SEB = Staphylococcal enterotoxin B, ^c^PHA = Phytohaemagglutinin. *Concentrations above or below the limit of quantification were extrapolated from the calibration curve.

Large inter-individual variability in cytokine concentrations was observed, even after age-adjustment (explaining only 3–7% of inter-individual variability), resulting in large coefficients of variation for all cytokines (Table 3). The unexplained residual variability (within-patient variability, including analytical error and model miss-specification) was 9.4% to 24.5%.

**Figure 3:**
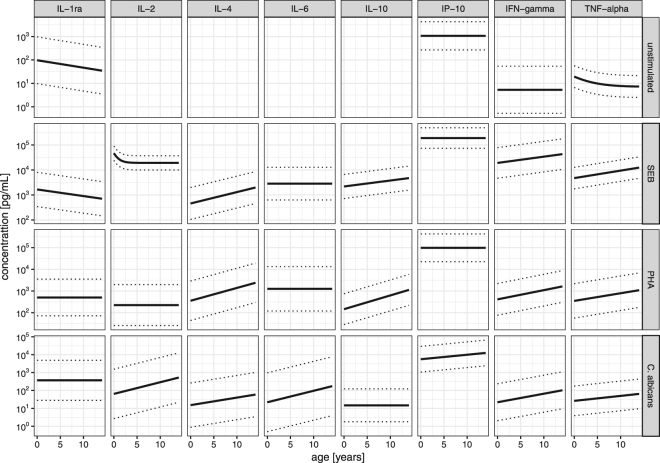
Cytokine for age percentile; solid line represents the 50^th^ and dotted lines the 5^th^ and 95^th^ percentile, respectively. Data are presented on a semi-logarithmic scale (log10-scale for cytokines, normal-scale for age). For cytokines 10^−2^ pg/mL equals a concentration of 0.01 pg/mL, 10^0^ equals 1 pg/mL, 10^2^ equals 100 pg/mL. Note that the depicted range of concentration differs between stimulants for better readability. For IL-2, IL-4, IL-6 and IL-10 percentiles for unstimulated samples were not quantifiable.

The cytokine for age percentiles were calculated sex independently. The predicted percentiles curves are shown in Fig. 3. In unstimulated and simulated samples, highest normal values were measured for IP-10, which were 10–1000 times higher compared to the other quantifiable cytokines. The interval between 5^th^ and 95^th^ percentile was dependent on both the cytokine measured and the stimulatory antigen used. Despite large variation of the cytokine concentrations age-dependency could clearly be identified.

## Discussion

The present study provides important information on the development of cytokine expression in childhood. To our knowledge this is one of the largest studies investigating cytokine profiles in healthy children and the first to provide age-specific percentiles for cytokines. Previous studies investigating cytokines in healthy children mostly included a smaller number of individuals^22–26^, measured fewer cytokines^27–29^ or included infants only^30–32^.

The present study analysed both stimulated and unstimulated samples as previous publications demonstrated that several cytokines are below the LLOQ in unstimulated conditions^26,28,33^. Indeed, our study showed that median concentrations for IL-2, IL-4, IL-6 and IL-10 were approximately 2 pg/ml in unstimulated blood and consequently not quantifiable in more than half of individuals. The importance, however, to separately analyse cytokine concentrations in unstimulated samples is highlighted by the fact that for other cytokines concentrations are well above the LLOQ and therefore further association such as age influence may be determined.

Indeed, TNF-α concentrations in unstimulated samples showed a significant age-associated decrease, particularly in the first few years of life. Similar findings were reported by a study including 79 children below the age of 5 years undergoing elective surgery, demonstrating higher TNF-α concentrations below two years of age compared to older children^28^. Equally, another study including 275 healthy children between 3 and 17 years of age also reported higher values in children compared to adults^33^.

In contrast to unstimulated samples, TNF-α concentrations in stimulated samples clearly show an age-associated increase. This finding is in line with several previous publications describing an age-associated increase of TNF-α release in different ethnic cohorts^12,13,29,34,35^. Two studies investigating intracellular cytokines found that TNF-α-producing T cells gradually increased in childhood and in one study this phenomenon was documented to continue even beyond 50 years of age^12,13^. It has been speculated that increasing TNF-α release with age results from the gradual development of the immune functionality and repertoire^34^. Interestingly, a further study showed that higher TNF-α concentrations are associated with an increased prevalence of autoimmune disease and atherosclerosis and furthermore a lower response to TNF-α antagonists has been found particularly in the elderly^36^. It remains however challenging to explain why non-stimulated TNF-α correlates negatively and stimulated TNF-α positively with age. One explanation might be the pleiotropic function of TNF-α and its complex homeostatic function^37^. TNF-α not only has pro-inflammatory and other immunological properties but plays a key role in the development of lymphoid organs and tolerance of macrophages all of which are of vital importance in early life^37^.

IL-1ra, another cytokine involved in homeostatic functions in children, shows very similar to TNF-α an age-associated decrease. This is in line with the findings from a study in which the authors hypothesized that the pattern observed is linked to a potential role of IL-1ra in bone development^33^. More recently, IL-1ra deficiency was described in the first few children, which is associated with skin pustulosis and bone deformity resulting from unopposed IL-1 signaling^38^. This strongly supports the suggestion that the observed age-association of IL-1ra in unstimulated samples is related to the physiologic bone development in childhood.

In stimulated samples, further cytokines are shown to have an age-associated increase. This is consistently seen across all stimulatory antigens particularly for IFN-γ and IL-4. An age-associated IFN-γ increase has been found in several previous studies including many different stimulatory antigens^12,13,22–24,29,32,35,39–41^. It is indeed one of the most consistent findings in previous studies investigating the development of cytokine release in children^42^ and it has been attributed to the reduced functionality and number of antigen presenting cells in early life^43^. Despite this compelling evidence of age influencing IFN-γ release current diagnostic tests using IFN-γ-associated read-outs such as IGRA do not have age-adapted cut-offs. It is therefore not surprising that IGRAs have an age-associated decrease in performance and are prone to false negative results in younger patients^44^.

IP-10, which is produced in response to IFN-γ, also showed an age-association in the present study, with higher concentrations in older children. This finding is in line with a previous Belgian study including infants and adults showing increasing IP-10 concentrations at 3 and 6 months of age^35^. Adult concentrations were reached as early as 9 months of age depending on the *in-vitro* stimulant used^35^. One of the challenges in measuring IP-10 are the high concentrations which were 10 to 1000 times higher than any other cytokine measured in the present study, requiring dilution for exact measurement. The high and therefore almost universally detectable concentration of this cytokine make it also an ideal candidate biomarker to be included in diagnostic tests, which is reflected by the numerous diseases in which it is currently investigated^45,42^.

The importance of age as a major factor influencing cytokine concentrations in a healthy paediatric population is further supported by the fact that sex, body weight and vitamin D concentration play only a minor role, as shown by fewer significant associations with cytokine concentrations. Some of those associations may even be confounded by a correlation with age, which was however weak for all three variables. Moreover, the large inter-individual variability in cytokine concentrations is also remarkable and further highlights that in addition to age up to 25% of the variability may be a result of the individuals’ personal immune response.

For comparison of results from our study with other studies it is important to acknowledge that age-specific percentiles will be best applicable to studies using similar *in-vitro* conditions. In addition, results from different assays and platforms to quantify cytokines have been shown to vary considerable and therefore absolute concentrations determined in this study are best comparable to those using the same assays and detection platform^46^. Despite this, it is likely that trends and patterns remain unchanged, as has been shown in a study comparing cytokine concentrations measured with four different kits on two platforms^46^.

A potential limitation of the study is that girls were underrepresented and therefore the influence of sex may be underestimated. It should also be kept in mind that girls were approximately two years older than boys as discussed above, and hence the observed sex effect might be introduced though a correlation with age and vice versa. The association of cytokine concentrations with vitamin D levels should also be interpreted with caution since vitamin D levels were higher in in younger children, likely as a result of standard supplementation recommendation in the first year of life. In addition, there were comparably fewer children in the first year of life included since parents were more reluctant for their children to take part in the study and blood sampling was more often unsuccessful. This might have impeded our ability to detect significant changes in concentrations within the narrow period of infancy/early childhood. We also acknowledge that the variability description for some of the cytokine concentrations may have been better characterized by alternative variability transformations (e.g. power transformations such as Box-Cox instead of log-transformation). Furthermore, the sample type, *in-vitro* stimulatory antigen and duration of stimulation are important factors potentially influencing cytokine concentrations and detectable patterns and therefore findings describing cytokine concentrations may not be applicable for other sample types or different *in-vitro* stimulatory conditions.

In summary, the results confirm the concept of age-association for cytokine release and highlight the complexity of immune maturation in childhood. Despite large inter-individual variation of cytokine concentrations age was identified as the strongest influencing factor among the investigated demographic and laboratory variables. Age-specific normal values therefore need to be considered for cytokine-based diagnostic purposes. In addition, these findings are relevant for the development of novel cytokine-based diagnostic tools and for optimal dosing of therapeutic agents targeting cytokines in childhood.

## Electronic supplementary material

Supplementary Information
