# Supplementary material for: Cytokine profiling in healthy children shows association of age with cytokine concentrations
Source: Sci Rep. 2017 Dec 19;7:17842. doi: 10.1038/s41598-017-17865-2 (PMC5736560; doi:10.1038/s41598-017-17865-2)
Supplement: Supplementary file 1 — Supplementary Information [file 41598_2017_17865_MOESM1_ESM.doc]

**Supplemental Material**

**Manuscript title: Cytokine profiling in healthy children shows association of age with cytokine concentrations**

**Author list**: Marie-Luise Decker^1,2^, Verena Gotta^2^, Sven Wellmann^3^, *Nicole Ritz^1,2^

^1^Infectious Diseases and Vaccinology Unit, University of Basel Children’s Hospital, 4056 Basel, Switzerland

^2^Paediatric Pharmacology and Pharmacometrics, University of Basel Children’s Hospital, 4056 Basel, Switzerland

^3^Neonatology Unit, University of Basel Children’s Hospital (UKBB), 4056 Basel, Switzerland

**Supplemental material**

**Box 1**: Linear regression model for associations of cytokine concentrations (log-transformed)with anthropometric and other parameters. In the equation y is the dependent variable (cytokine concentration), β_0_ is the intercept (baseline or reference concentration (pg/mL) in infancy), β_1_ the slope (≈fractional change in cytokine levels with one unit of the independent variable x_1_).

| 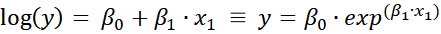 |
| --- |

**Boxes 2 a, b, c:** Description of mixed-effect regression for each condition and cytokine: **a)** tested structural models for assessment of alternative relationships of cytokine concentrations with age; **b)** individual-specific random-effect model; **c)** residual error model.

a)

| General model: 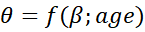  Model 1 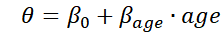  Model 2 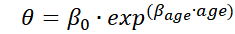  Model 3 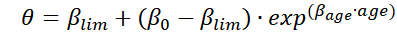  Model 4 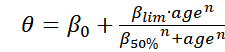 |
| --- |

In the equation
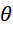
 is the typical age-specific cytokine concentration under a given condition, β_0_ is the respective typical baseline concentration (pg/mL) for a newborn (age = 0 years), β_age_ is the typical slope characterizing a linear change with age (pg/mL per 1 year of age) in model 1, and an exponential change with age (≈fractional change per 1 year of age) in models 2 and 3. In model 3 a maximal physiologic change over the studied age-range is assumed, with an upper or lower limit β_lim_ (pg/mL). Model 4 describes a logistic (hyperbolic or sigmoidal) function with a maximal change β_lim_ (pg/mL), with β_50%_ (years) representing the age at which a half-maximal change is observed, and where *n* is the hill coefficient determining the steepness of change around β_50%_. The Akaike Information Criterion (AIC) was used for model selection, which was calculated based on the NONMEM objective function value (OFV) corresponding to minus-2-times log-likelihood (AIC = OF + 2 number of model parameters).

b)

| 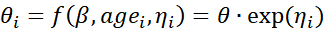 with 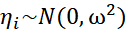 |
| --- |

Where
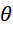
 denotes the typical age-specific cytokine value in the population,
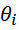
 the individual age-specific cytokine value, and where
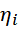
 denotes the individual random-effect, that was assumed to be normally distributed with variance
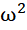
 and mean 0.

**c)**

| 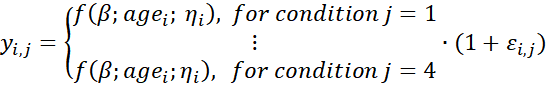, with 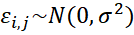 |
| --- |

Where
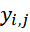
 is the observation under condition j of the i^th^ individual,
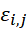
is the proportional residual error (representing mainly assay variability), that was assumed to be normally distributed with variance
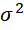
and mean 0.

**Box 3**: Equation for the calculation of 5^th^ and 95 ^th^ percentiles from estimated inter-individual variability of mixed effect regression.
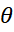
 = typical age-specific cytokine concentration (mean at the log-scale),
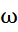
 = standard deviation of estimated between-subject variability
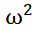
.


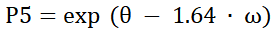


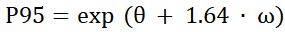


**Table 1:** Parameter estimates of the censored mixed effect linear and non-linear regression analysis. Confidence intervals of estimates are given in brackets (calculated by ±1.96 ∙ standard error).

**In most cases, in which a significant age effect was estimated, structural model 2 best described the cytokine change over age, with β_0_: baseline concentration (pg/mL) for a newborn (age = 0 years) and β_age_: slope characterizing an exponential change with age (≈fractional change per 1 year of age). Only for IL-2 (after SEB stimulation) and TNF-α (unstimulated) structural model 3 improved the description of cytokine change over age, with additional parameter β_lim_: upper limit corresponding to a maximal physiologic change over the studied age-range (pg/mL). If no age effect could be estimated, β_0_ corresponds to the geometric mean cytokine concentration.
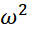
 variance of inter-patient variabililty (CV_ω_: corresponding coefficient of variation (%) =
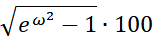
). CV_ε_: coefficient of variation (%) of proportional residual error =
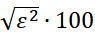
.**

| **Cytokine** | **Unstimulated** | | | **SEB** | | | **PHA** | | | **Candida** | | | **Residual error** |
| --- | --- | --- | --- | --- | --- | --- | --- | --- | --- | --- | --- | --- | --- |
|  | **β_0_ (β_lim_)**  [pg/mL] | **β_age_**  [1/year] | 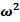 **(CV_ω_)** | **β_0_ (β_lim_)**  [pg/mL] | **β_age_**  [1/year] | 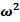 **(CV_ω_)** | **β_0_ (β_lim_)**  [pg/mL] | **β_age_**  [1/year] | 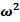 **(CV_ω_)** | **β_0_ (β_lim_)**  [pg/mL] | **β_age_**  [1/year] | 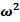 **(CV_ω_)** | **CV_ε_**  [%] |
| **IL-1ra** | 97 | -0.074 | 1.39 (244%) | 1670 | -0.061 | 0.96 (123%) | 505 | - ^b^ | 1.2 (175%) | 372 | - ^b^ | 1.57 (327%) | 9.4 |
|  | [66; 128] | [-0.123;-0.024] | [1.27;1.51] | [1178; 2154] | [-0.105;-0.017] | [0.87 ;1.04] | [431; 580] |  | [1.08;1.28] | [304; 440] |  | [1.43 ;1.70] | [6.3; 12.4] |
| **IL-2** |  | X^a^ |  | 46'800 **(19'300)** | -1.13 | 0.40 (42%) | 225 | - ^b^ | 1.32 (217%) | 64.8 | 0.15 | 1.93 (641%) | 24.5% |
|  |  |  |  | [20327; 73341]  ([17958; 20644]) | [-0.18; -0.43] | [0.32;0.47] | [188; 262] |  | [1.19;1.43] | [30.3; 99.2] | [0.070; 0.230] | [1.75;2.10] | [18.7; 30.4%] |
| **IL-4** |  | X^a^ |  | 458 | 0.105 | 0.89 (111%) | 358 | 0.136 | 1.27 (201%) | 15.2 | 0.097 | 1.73 (437%) | 14.5 |
|  |  |  |  | [316; 600] | [0.062; 0.149] | [0.80;0.98] | [205; 510] | [0.072; 0.199] | [1.09;1.43] | [4.5; 26.0] | [0.011; 0.183] | [1.39;2.02] | [-9.7; 38.6] |
| **IL-6** |  | X^a^ |  | 2850 | - ^b^ | 0.92 (114%) | 1260 | - ^b^ | 1.43 (260%) | 22 | 0.148 | 2.30 (1412%) | 12.9 |
|  |  |  |  | [2534; 3160] |  | [0.83;0.99] | [1045; 1481] |  | [1.30;1.55] | [13.7; 30.3] | [0.084; 0.212] | [2.09;2.50] | [9.1; 16.6] |
| **IL-10^c^** |  | X^a^ |  | 2190 | 0.055 | 0.67 (76%) | 146 | 0.146 | 1.0 (130%) | 14.8 | - ^b^ | 1.29 (206%) | 12.6 |
|  |  |  |  | [1812; 2575] | [0.029; 0.081] | [0.61 ;0.73] | [106; 186] | [0.105; 0.188] | [0.91;1.08] | [12.3; 17.2] |  | [1.16 ;1.40] | [9.1; 16.1] |
| **IP-10** | 1070 | -^b^ | 0.84 (101%) | 190’000 | - ^b^ | 0.57 (62%) | 98’000 | - ^b^ | 0.89 (111%) | 5600 | 0.058 | 1.01 (132%) | 16.5 |
|  | [961; 1181] |  | [0.76;0.91] | [176694; 203572] |  | [0.52;0.63] | [87616; 108330] |  | [0.81;0.97] | [4313; 6889] | [0.022; 0.093] | [0.91;1.09] | [13.3; 19.8] |
| **INF-γ** | 5.4 | - ^b^ | 1.40 (248%) | 19’200 | 0.058 | 0.86 (104%) | 413 | 0.098 | 1.02 (135%) | 22 | 0.11 | 1.45 (266%) | 21.1 |
|  | [4.1; 6.6] |  | [1.23 ;1.56] | [14992; 23402] | [0.025; 0.092] | [0.78 ;0.93] | [305; 521] | [0.059; 0.138] | [0.93;1.11] | [14; 30] | [0.054; 0.166] | [1.31 ;1.57] | [17.2; 24.9] |
| **TNF-α** | 19.5 (**7.1**) | -0.3 | 0.65 (73%) | 4740 | 0.069 | 0.60 (66%) | 352 | 0.081 | 1.11 (157%) | 26.3 | 0.064 | 1.16 (169%) | 17.7 |
|  | [13.4; 25.5]  ([5.2 9.1]) | [-0.5; -0.1] | [0.58 ;0.71] | [3960; 5530] | [0.044; 0.094] | [0.53 ;0.67] | [254; 450] | [0.038; 0.124] | [1.01;1.21] | [17.7; 35.0] | [0.014; 0.114] | [1.05 ;1.26] | [13.6; 21.8] |

^a^X: no regression analysis performed since > 50% of data below limit of quantification.

^b^ -: no significant change with age estimated (p>0.05 for additional parameter β_age_.

**^c^**one individual had extremely low IL-10 levels and was excluded for estimation of the variability. This did not have an influence on the estimated age-relationship.
